# Health resource allocation in Western China from 2014 to 2018

**DOI:** 10.1186/s13690-023-01046-x

**Published:** 2023-02-22

**Authors:** Zheng Wang, Haoyu He, Xi Liu, Hongkuang Wei, Qiming Feng, Bo Wei

**Affiliations:** 1 School of Information and Management, Guangxi Medical University, 22 Shuangyong Road, Nanning, 530021 Guangxi China; 2 Guangxi Medical College, 8 Kunlun Road, Nanning, 530000 Guangxi China; 3 Quality Management Department, the Affiliated Hospital of Stomatology, Guangxi Medical University, 10 Shuangyong Road, Nanning, 530021 Guangxi China; 4 Maternal and Child Health Care Hospital of Guangxi Zhuang Autonomous Region, 225 Xinyang Road, Nanning, 530002 Guangxi China

**Keywords:** Resource allocation, Regional health planning, Health equity

## Abstract

**Background:**

Health equity has persistently been a global concern. How to fairly and appropriately allocate health resources is a research hotspot. While Western China is relatively backward economically and presents difficulties for the allocation of health resources, little attention has been given to the equity of resource allocation there. This study analysed the equity of allocation of beds, physicians and nurses in Western China from 2014-2018 to provide targeted guidance for improving the equity of health resource allocation.

**Methods:**

Data for 2014-2018 obtained from the Statistical Yearbook (2015-2019) of provinces (autonomous regions and municipalities) were used to analyse health resource allocation in terms of beds, physicians and nurses in Western China. The Lorenz curve and Gini coefficient were calculated to evaluate equity in the population dimension and geographic dimension. The Theil index was used to measure the inequity of the three indicators between minority and nonminority areas.

**Results:**

The number of beds, physicians and nurses in Western China showed an increasing trend from 2014-2018. The Lorenz curve had a smaller curvature in the population dimension than in the geographic dimension. The Gini coefficients for health resources in the population dimension ranged from 0.044 to 0.079, and in the geographic dimension, the Gini coefficients ranged between 0.614 and 0.647. The above results showed that the equity of health resource allocation was better in the population dimension than in the geographic dimension. The Theil index ranged from 0.000 to 0.004 in the population dimension and from 0.095 to 0.326 in the geographic dimension, indicating that the inequity in health resource allocation was higher in the geographic dimension. The intergroup contribution ratios of the Theil index in both the population and geographic dimensions were greater than 60%, indicating that the inequity in resource allocation was mainly caused by intergroup differences, namely, the allocation of health resources within the province. Among them, the inequity of physicians and nurses allocation was the most obvious.

**Conclusions:**

From 2014 to 2018, the total amount of health resources have improved in Western China. However, health resource allocation in Western China was still inequitable in the population and geographic dimensions, and the inequity of health resource allocation in the geographic dimension showed a tendency to worsen. Meanwhile, although the inequity of human recourse allocation was alleviated in 2018 compare with 2014, the inequity of human resource distribution within provinces was still obvious. The government can increase the number of health resources and improve the accessibility of health resources by increasing financial investment, strengthening humanistic care for health workers, and establishing internet hospitals.

## Background

The concept of health equity was first proposed in the United Kingdom, where the government’s Health Inequalities Panel described social health disparities and attributed disparities to differences in the socioeconomic environment [1]. Health equity includes equity of health financing, equity of health service utilization and equity of health results. Health resource distribution is one of the components of equity in health service utilization.

Equity in health resource allocation refers to the way in which health resources are distributed and flow between health care sectors or regions, it can reflect the degree of health equity and is a significant aspect of social equity [2]. The allocation of health resources is also recognized as the basis for the sustainable development of health undertakings, and it also plays an important role in ensuring that the performance of the health system reaches the standard. The fairness of health resource allocation, as the premise of the fairness of the utilization of health services, is causally related to the health rights of the people [3–5]. Health resource allocation equity directly affects health equity and is one of the conditions for realizing health equity. Hence, health resource allocation equity has attracted attention worldwide [6].

China also attaches great importance to the fair allocation of resources. In the *Outline of the Healthy China 2030 Plan* released in 2016, it is clearly proposed to "gradually reduce the differences in basic health services and health levels between urban and rural areas, regions and groups of people, achieve universal health coverage and promote social health equity". Especially after the landmark Basic Medical Care and Health Promotion Law was promulgated by the Standing Committee of the National People's Congress of China on December 28, 2019 [7], researchers hoped that access to health services would be promoted. Many researchers have analysed the equity of health resources in provinces and cities by using a combination of various analytical methods. For instance, Ren Zhenghong et al. analysed the distribution of human resources in obstetrics and gynaecology in China [8]. Sun Jian et al. analysed the equity and efficiency of health resource allocation and health service utilization in China [9]. These studies have provided strong support to policy makers, however, few studies have focused on Western China.

Western China includes 12 provinces (autonomous regions and municipalities), namely, Shaanxi, Sichuan, Yunnan, Guizhou, Gansu, Qinghai, Guangxi Zhuang Autonomous Region, Ningxia Hui Autonomous Region, Tibet Autonomous Region, Xinjiang Uygur Autonomous Region, Inner Mongolia Autonomous Region and Chongqing Municipality. By the end of 2018, the area of Western China accounted for 70.6% of the country. The population was 379,558,700, accounting for 27.2% of the total population. Western China is a less-developed region, its economy is relatively backward compared to that of other regions, and its terrain is mostly mountains, plateaus, etc., which makes transportation difficult. These factors increase the difficulty of allocating health resources, which may affect the health level of people in Western China. Although Western China accounts for more than two-thirds of the country's area. The number of physicians in Western China in 2018 was 874,160, lower than the 1,650,531 in Eastern China and 1,048,786 in the central region, and the equity of physician allocation was lower than that in other developed regions, according to previous studies [10–12]. This indicates that more attention should be given to the specific situation of health resource allocation in Western China so that the equity of health resource allocation can be improved. However, few studies have focused on the equity allocation of health resources in Western China.

Beds, physicians and nurses are important components of health resources and determine patients' accessibility to health care. Their allocation is directly related to whether health care is equal [13, 14]. This study aims to analyse the allocation equity of beds, physicians and nurses in Western China from 2014-2018 and to propose corresponding countermeasures and suggestions based on the analysis results to further promote the equity of health resource allocation in Western China.

## Data sources and methods

### Data sources

The health resource indicators in this study are the number of beds, physicians and nurses. Table 1 provides the definition and measurement of the three indicators. The data of these three indicators from 2014-2018 were collected from the Statistical Yearbook (2015-2019) of each province, which was obtained from the statistical office websites of each province. The sources of all data were authoritative and reliable, so the data were applied directly in this study without using inclusion or exclusion criteria.

**Table 1:** Indicators of health resource allocation, their definitions, and how they were measured

**Indicator**	**Definition**	**How indicator was measured**
Beds	It refers to the actual number of beds in medical institutions, including formal beds, simple beds, nursing beds, and beds under sterilization or repair. Neonatal beds, prenatal beds, observation beds, temporary beds and beds for patients' families are excluded.	Number of beds/total population× 1000
physicians	It refers to the physicians who hold a practising physician certificate, including practising physicians and assistants in China. Those involved in the management of health workers as part of the health workforce, such as presidents, vice presidents and party secretaries, are excluded.	Number of physicians/total population× 1000
nurses	It refers to registered nurses who have obtained a legal practising nurse certificate. Those engaged in the management of health workers, such as presidents, vice presidents, and party secretaries, are not included in the health workforce.	Number of nurses/total population×1000

### Functional regional division

Western China contains several ethnic minority autonomous regions. The number of ethnic minorities in these regions is relatively large. Influenced by factors such as religious culture, folk medicine and economic culture, people's health behaviour and health resource allocation may be significantly different in comparison with other regions [15]. Therefore, this study divides Western China into ethnic minority and nonminority regions to explore whether there were differences in resource allocation equity between these two regions. The ethnic minority regions include the Guangxi Zhuang Autonomous Region, Ningxia Hui Autonomous Region, Tibet Autonomous Region, Xinjiang Uygur Autonomous Region and Inner Mongolia Autonomous Region. The nonminority regions include Shaanxi, Sichuan, Yunnan, Guizhou, Gansu, Qinghai and Chongqing.

### Measurements of inequity

In analysing the equity of health resource allocation, the Lorenz curve, Gini coefficient and Theil index have their own advantages and disadvantages. By using these analysis methods together, the equity of health resource allocation can be analysed objectively, and the main factors affecting the inequity of resource allocation can be found. Therefore, in this paper, we used the above three methods together to analyse the equity of health resource allocation in Western China in the demographic dimension and the geographic dimension.

### Lorenz curve

In the early period, the Lorenz curve was mainly used to evaluate the equity of resource or income distribution and measure the equity difference. Currently, it is commonly used to measure the equity of health care resources [16]. When using the Lorenz curve to measure health resource equity, the population dimension and geographic dimension can be analysed after dividing the income or resources of residents by different groups or regions. To analyse the population dimension, a coordinate system is established with the cumulative percentage of the population and the cumulative percentage of each health resource as coordinates. To analyse the geographic dimension, a coordinate system is established based on the cumulative percentage of land area and the cumulative percentage of each health resource. Then, a line of absolute fairness is drawn by connecting the zero point and each coordinate point. According to the Lorenz curve, fairness is judged on the basis of the distance from the absolute fairness line. The closer a point is to the absolute fairness line, the more fair it is, and vice versa [17].

### Gini coefficient

The Lorenz curve can evaluate the equity of resources, but it cannot quantify the specific difference [18]. Therefore, the Gini coefficient is often calculated through the Lorenz curve to indicate the fairness of the distribution of social resources or property [19] :

1 $$G=\frac{1}{{2n}^{2}\mu }\sum_{i-1}^{n}\sum_{j-1}^{n}\left|{y}_{i}-{y}_{j}\right|$$

where $G$ stands for the Gini coefficient, n for the group number,* μ* for the resident income, and ${y}_{i}$ and ${y}_{j}$ for the per capita income of groups* i* and *j* [20].

The Gini coefficient takes a range of [0, 1]; the closer to 0, the more equitable it is; the closer to 1, the less equitable it is. It is generally believed that a Gini coefficient less than 0.3 means that the resource allocation is in the most fair state; 0.3-0.4 is the normal state; a level above 0.4 warns of unfairness; and a level above 0.6 indicates a dangerous state of high unfairness [21, 22].

### Theil index

The Theil index is the major instrument used to measure fairness, and it can directly reflect the influence of different regions on resource allocation. Its value range is [0, 1], and the lower the value of the Theil index is, the better the balance of social resource allocation in the region [23, 24]. The Theil index can divide the overall variance into different groups and calculate the contribution ratio. The larger the contribution ratio is, the more significantly the resource allocation inequity is proven. Hence, when using the Theil index to analyse health resource allocation, it is also possible to measure the fairness of intragroup and intergroup health resource allocation. In this study, the intragroup refers to the comparison between ethnic minority and nonminority regions, and the intergroup refers to the comparison of resource allocation within each province.

The calculation formula of the Theil index is as follows [25]:

2 $$Theil-l=\sum_{i-1}^{n}{P}_{n}{\mathrm{log}}_{\frac{{P}_{n}}{Yn}}$$

3 $${T}_{inter}=\sum_{g-1}^{k}{P}_{j}{\mathrm{log}}_{\frac{{P}_{j}}{Yj}}$$

4 $${T}_{intra}=\sum_{g-1}^{k}{P}_{j}{T}_{j}$$

5 $$Theil={T}_{inter}+{T}_{intra}$$

where ${P}_{n}$ refers to the proportion of the population of each province in the total population; ${P}_{j}$ is the proportion of the population of each region in the total population; and each ${y}_{n}$ is the proportion of resources of each province in the total number of resources on this dimension. ${y}_{j}$ shows the proportion of resources of each region in the total number of resources [26, 27]

## Results

### Basic situation of health resource allocation in Western China from 2014 to 2018

Tables 2–3 exhibit the distribution of health resources in Western China from 2014 to 2018. The three indicators showed an upward trend. Based on the distribution of health resources in 2018, Xinjiang had the most beds per 1,000 people, 7.19, followed by Sichuan (7.18) and Chongqing (7.10), while Tibet had the fewest beds per thousand people (4.88), followed by Guangxi (5.20) and Ningxia (5.96). Inner Mongolia had the highest number of physicians per 1,000 people (2.90), followed by Ningxia (2.82) and Qinghai (2.68), while Yunnan had the lowest number, 2.06, followed by Guangxi (2.15) and Guizhou (2.26). Shaanxi had the most nurses per 1,000 people (3.57), followed by Ningxia (3.38) and Chongqing (3.07), while Tibet had the least (1.62), followed by Gansu (2.44) and Yunnan (2.83).

**Table 2:** The distribution of beds per 1,000 population in Western China from 2014-2018

**Region**	2014	2015	2016	2017	2018
**Guangxi**	4.42	4.47	4.46	4.94	5.20
**Shaanxi**	5.28	5.59	5.91	6.29	6.57
**Gansu**	4.72	4.91	5.15	5.58	6.17
**Qinghai**	5.66	5.87	5.86	6.41	6.49
**Ningxia**	4.91	5.06	5.38	5.84	5.96
**Xinjiang**	6.22	6.37	6.54	6.85	7.19
**Sichuan**	5.65	5.96	6.28	6.79	7.18
**Chongqing**	5.37	5.85	6.26	6.71	7.10
**Yunnan**	4.77	5.01	5.31	5.72	6.03
**Guizhou**	5.19	5.57	5.92	6.51	6.82
**Inner Mongolia**	5.15	5.33	5.53	5.94	6.27
**Tibet**	3.75	4.33	4.37	4.78	4.88

**Table 3:** Distribution of human resources per 1,000 population in Western China from 2014 to 2018

**Region**	**2014**		**2015**		**2016**		**2017**		**2018**	
	physicians	nurses	physicians	nurses	physicians	nurses	physicians	nurses	physicians	nurses
**Guangxi**	1.82	2.19	1.90	2.40	2.00	2.53	2.07	2.70	2.15	2.85
**Shaanxi**	2.03	2.58	2.10	2.80	2.25	3.06	2.43	3.31	2.56	3.57
**Gansu**	1.84	1.75	1.90	1.80	2.02	1.94	2.14	2.23	2.26	2.44
**Qinghai**	2.22	2.19	2.30	2.20	2.30	2.42	2.59	2.76	2.68	2.91
**Ningxia**	2.27	2.28	2.40	2.40	2.53	2.68	2.67	3.16	2.82	3.38
**Xinjiang**	2.38	2.60	2.40	2.70	2.51	2.82	2.55	2.89	2.55	2.91
**Sichuan**	2.21	2.16	2.20	2.30	2.24	2.51	2.35	2.75	2.46	2.96
**Chongqing**	1.94	1.32	2.00	2.30	2.12	2.54	2.23	2.76	2.46	3.07
**Yunnan**	1.60	1.23	1.70	2.00	1.80	2.22	1.96	2.68	2.06	2.83
**Guizhou**	1.65	1.92	1.80	2.20	1.94	2.42	2.11	2.74	2.26	3.03
**Inner Mongolia**	2.48	2.26	2.60	1.90	2.63	2.64	2.78	2.84	2.90	3.02
**Tibet**	1.76	1.43	1.90	1.00	1.98	1.16	2.26	1.32	2.42	1.62

### Lorenz curve of health resources in Western China from 2014 to 2018

**Fig. 1:**
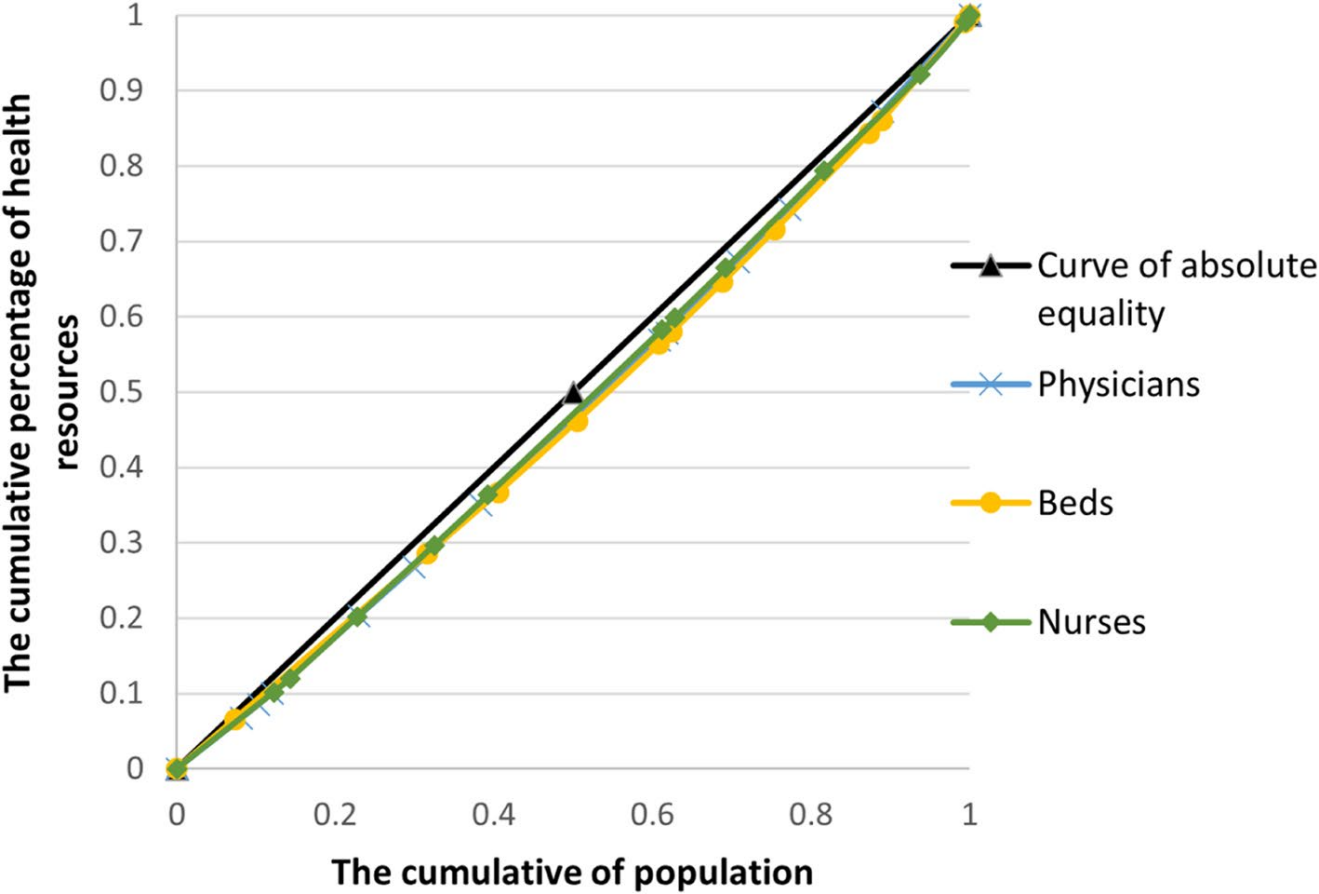
Lorenz curve of health resource distribution in the population dimension in Western China from 2014 to 2018

**Fig. 2:**
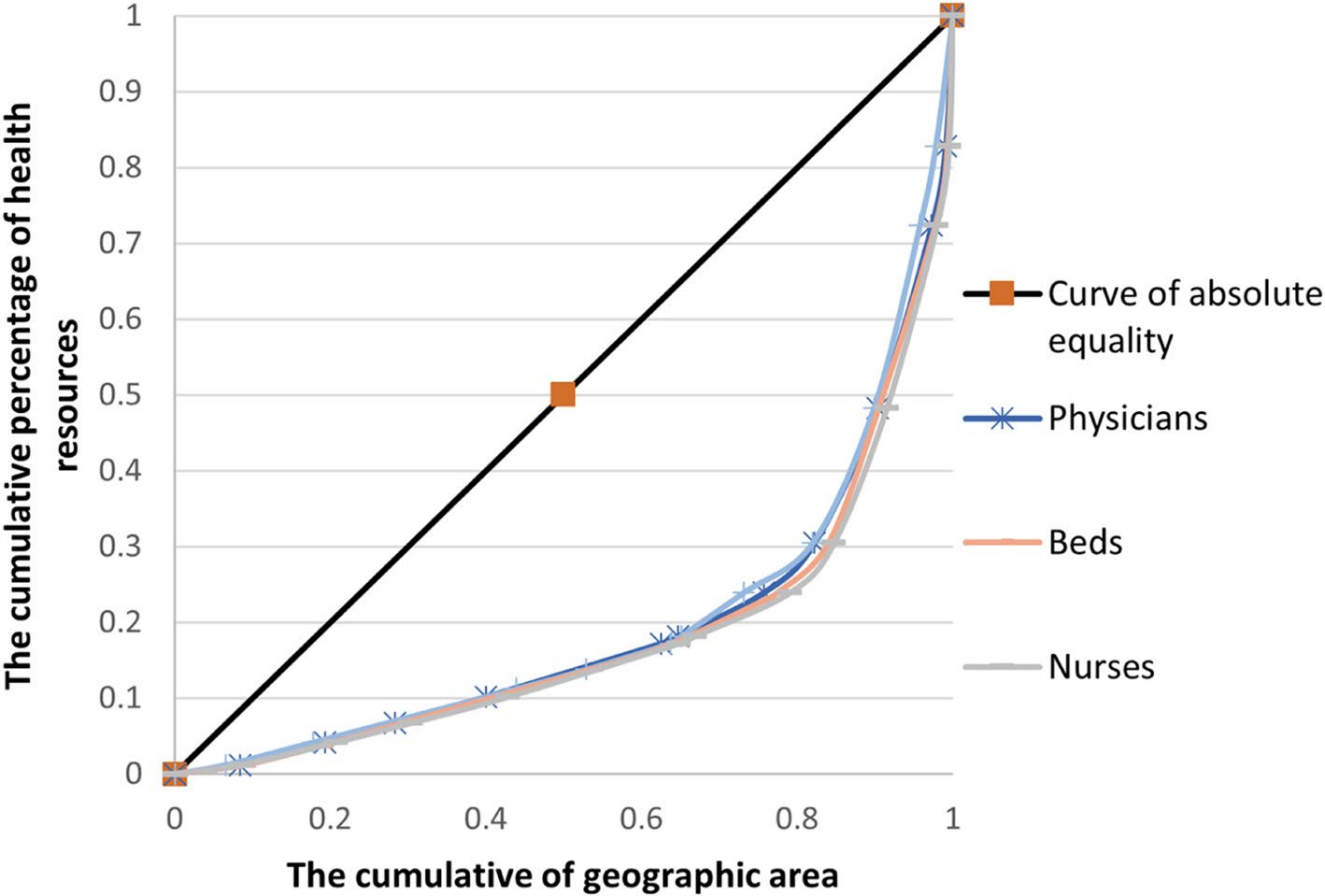
Lorenz curve of health resource distribution in the geographic dimension in Western China from 2014 to 2018

Taking the cumulative population ratio as the X-axis and the cumulative percentage of health resources as the Y-axis, the Lorenz curve under the population dimension was plotted, and the result is shown in Fig. 1. Similarly, the Lorenz curve under the geographic dimension was drawn with the geographical area as the X-axis and the cumulative percentage of health resources as the Y-axis. The results are shown in Fig. 2. Comparing the Lorenz curves in the two dimensions, the curve in the geographic dimension is significantly more distant from the absolute fairness line than that in the population dimension, which indicates that the allocation of health resources in the population dimension is better than that in the geographic dimension. Moreover, the curvatures of the Lorenz curves of the three analysis indicators in the population dimension are similar, indicating that the difference in equity degree is small. The Lorenz curves of the three analysis indicators in the geographic dimension show similar results.

### Gini coefficient of health resources in western China from 2014 to 2018

Gini coefficients were calculated according to the Lorenz curve results of the population and geographic dimensions, as shown in Table 4. In 2014, the three analysis indicators of health resources in the population dimension were between 0.058 and 0.078, less than 0.2, which indicates a state of high equity. The Gini coefficients of the three health resources analysis indicators in the geographic dimension were between 0.615 and 0.636, higher than 0.6, showing a highly unfair state.

**Table 4:** Gini coefficients of health resources in different dimensions in Western China from 2014 to 2018

	**Population dimension**			**Geographical dimension**		
**Year**	**beds**	**physicians**	**nurses**	**beds**	**physicians**	**nurses**
**2014**	0.0583	0.0785	0.0719	0.6275	0.6151	0.6359
**2015**	0.0577	0.0694	0.0644	0.6300	0.6136	0.6388
**2016**	0.0584	0.0601	0.0611	0.6338	0.6158	0.6421
**2017**	0.0580	0.0578	0.0438	0.6344	0.6149	0.6433
**2018**	0.0574	0.0525	0.0462	0.6343	0.6200	0.6469

In 2018, the three analysis indicators of health resources in the population dimension were between 0.046 and 0.057, less than 0.2, which indicates the most fairness. The Gini coefficients of the three health resources analysis indicators in the geographic dimension were between 0.620 and 0.647, higher than 0.6, showing a highly unfair state. Comparing the Gini coefficients in 2014 and 2018, the equity of health resource allocation in the population dimension was better than that in the geographic dimension. Furthermore, the three analysis indicators had similar values in each dimension, indicating that the fairness difference in each dimension was small. This verifies the results of the Lorenz curve drawing.

**Fig. 3:**
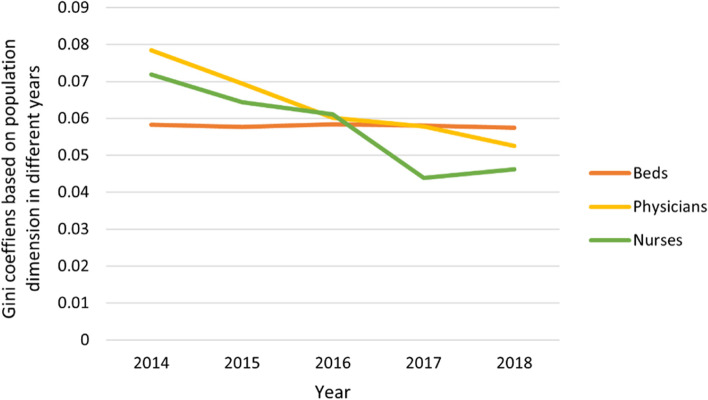
Gini coefficient variation tendency of health resources in the population dimension in Western China from 2014 to 2018

**Fig. 4:**
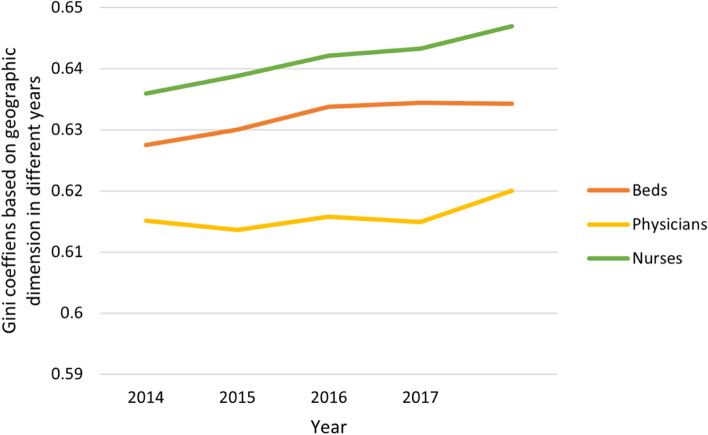
Gini coefficient variation tendency of health resources in the geographic dimension in Western China from 2014 to 2018

In addition, Fig. 3 shows that the Gini coefficient in the population dimension presents a downward trend, which indicates that equity improved. Moreover, the Gini coefficient on the basis of geographic area presents an overall gradual upward trend, as shown in Fig. 4.

### Theil index of health resources in western China

Based on the Theil index analysis of different dimensions shown in Table 5, the Theil index in the population dimension is lower than that in the geographic dimension, which indicates that the allocation of health resources is better in the population dimension. Moreover, the analysis results of the Theil index contribution ratios of health resource allocation from the two dimensions in Table 6 show higher intergroup equity than intragroup equity. From the perspective of the population dimension, the three indexes all show that the intergroup contribution ratios was greater than the intragroup contribution ratios, and the contribution rates all exceeded 60%. Among them, the contribution ratios of physicians and nurses in the population dimension was the most obvious. The above results indicate that the inequity of resource allocation mainly comes from intergroup comparison, namely, inside the provinces, and the inequitable allocation of human resources (physicians and nurses) is obvious. The contribution ratios in the geographic dimension showed a similar tendency, and the rate exceeded 60%.

**Table 5:** Theil index of health resources in two dimensions in Western China from 2014-2018

Dimensions	Year	Beds			Physicians			Nurses		
		T	T_intra_	T_inter_	T	T_intra_	T_inter_	T	T_intra_	T_inter_
**Population**	**2014**	0.002	0.002	0.000^a^	0.004	0.004	0.000	0.004	0.004	0.000
	**2015**	0.002	0.002	0.000	0.003	0.003	0.000	0.003	0.003	0.000
	**2016**	0.002	0.002	0.000	0.003	0.002	0.000	0.003	0.003	0.000
	**2017**	0.002	0.002	0.001	0.002	0.002	0.000	0.002	0.002	0.000
	**2018**	0.002	0.002	0.001	0.002	0.002	0.000	0.002	0.002	0.000
**Geographical**	**2014**	0.318	0.200	0.117	0.300	0.204	0.096	0.228	0.097	0.228
	**2015**	0.321	0.202	0.119	0.299	0.204	0.095	0.230	0.099	0.230
	**2016**	0.326	0.205	0.121	0.301	0.206	0.096	0.231	0.102	0.231
	**2017**	0.326	0.204	0.122	0.300	0.202	0.098	0.227	0.108	0.227
	**2018**	0.326	0.203	0.123	0.306	0.205	0.101	0.227	0.111	0.227

^a^Since there are only three decimal places to the right of the decimal point, there are several Theil index values of 0.000 in the table

**Table 6:** Contribution ratios of health resources in two dimensions in Western China from 2014-2018

Dimensions	Year	Beds		Physicians		Nurses	
		T_intra_	T_inter_	T_intra_	T_inter_	T_intra_	T_inter_
**Population**	**2014**	8.00%	92.00%	10.31%	89.69%	8.82%	91.18%
	**2015**	12.53%	87.47%	14.15%	85.85%	6.90%	93.10%
	**2016**	18.68%	81.32%	15.69%	84.31%	2.96%	97.04%
	**2017**	23.72%	76.28%	10.68%	89.32%	0.12%	99.88%
	**2018**	26.75%	73.25%	4.18%	95.82%	2.57%	97.43%
**Geographical**	**2014**	36.95%	63.05%	32.04%	67.96%	29.87%	70.13%
	**2015**	36.99%	63.01%	31.80%	68.20%	30.01%	69.99%
	**2016**	37.21%	62.79%	31.77%	68.23%	30.55%	69.45%
	**2017**	37.55%	62.45%	32.57%	67.43%	32.26%	67.74%
	**2018**	37.71%	62.29%	33.09%	66.91%	32.85%	67.15%

## Discussion

This study analysed the equity of bed, physician and nurse allocation in Western China from 2014-2018 by using the Lorenz curve, Gini coefficient and Theil index. The study found that health resource allocation in Western China was still inequitable in the population and geographic dimensions, and the inequity of health resource allocation in the geographic dimension showed a tendency to worsen. The inequitable allocation of health resources in Western China mainly came from the inequitable allocation of resources within provinces. Among them, the allocation of doctors and nurses is the most inequitable.

During 2014-2018, the number of beds, physicians and registered nurses in Western China showed an increasing trend. This indicates that the Chinese government measures have been effective in increasing health resources in Western China. The main purpose of the *China's 13th Five-Year Plan for Health and Wellness* (*13th Five-Year Plan*) is to plan the development of Chinese health services from 2015-2020, so the goals of the *13th Five-Year Plan* are still applicable to the period of this study (2014-2018). The *13th Five-Year Plan* states that the number of beds in medical institutions per 1,000 people should be 6, the number of physicians should be 2.5, and the number of nurses should be 3.14 in 2020 [28]. In 2018, the number of medical beds per 1,000 population in Western China basically met the planning expectations, except in Guangxi and Tibet. Only Shanxi, Qinghai, Ningxia, Xinjiang and Inner Mongolia met the number of physicians per 1,000 population as expected in the *13th Five-Year Plan*. The distribution of nurses is even more serious, with only Shaanxi and Ningxia Provinces having more than 3.16 nurses per 1,000 people. Thus, considering the goals of the *13th Five-Year Plan*, further improvement is still needed. It is recommended that the government strengthen its role in allocating health resources, enhance the feasibility of policies, strengthen relevant safeguard measures, ensure the implementation of relevant policies to improve the total amount of health resources in Western China, and further realize the goal of "health equity for all", which is emphasized in the *13th Five-Year Plan* and the *Outline of the Healthy China 2030 Plan* [29].

The results showed that the equity of health resource allocation in Western China was higher in the population dimension than under the geographic dimension, which may be because the current health resource allocation approach adopted in China is mainly based on population density [30, 31]. The results also show that the inequity of health resource allocation under the geographic dimension is increasing. This may be due to the complex geography of Western China, which has large geographical areas, high altitude, low population density (such as Xinjiang, Inner Mongolia and Tibet) and mostly mountains that make traffic difficult (such as Guangxi, Guizhou and Chongqing). The World Health Organization (WHO) recommends that everyone should have access to affordable, quality health care. However, the less equitable geographic distribution in Western China will lead to low access to services and low utilization of resources, which is inconsistent with the WHO's goal of universal health coverage and affects the health equity of the population [32]. The government must therefore improve the accessibility of health resources in Western China through a variety of approaches, such as hierarchical diagnosis and treatment systems, medical treatment alliances, and increased financial investment, to ensure people receive health resources timely and effectively [33–35].

Geographic information systems can also be used to support the allocation of health resources and further improve the accessibility of health resources. In addition, due to the geographical characteristics of Western China, it is not economically efficient to rely only on investment in equipment and capital to improve the accessibility of health resources. Therefore, the government can consider drawing on the experience of other countries, such as the United States and Japan, to establish internet hospitals and provide online consultations as a way to improve the accessibility of health resources.

The results of the Theil index analysis indicated that the inequality of resource allocation was mainly caused by the unequal distribution of health resources within the provinces. This may be caused by China’s current resource allocation policies, and the different population densities in different cities can be one of the reasons. The unique geography of Western China exacerbates inequity in allocation. Some Western rural areas have extremely inconvenient transportation and are unreachable by vehicle, which is inconvenient for the construction and transportation of health resources. Moreover, health resource allocation is related to economic development [34], and the level of economic development among cities and counties can lead to the unequal distribution of health resources within provinces. Therefore, when formulating regional development plans, the government should consider the economic strength of different regions and make targeted policies to reduce the inequity in human resource allocation caused by economic reasons.

We found that inequity in the intergroup distribution of human resources (physicians and nurses) was more pronounced in the demographic and geographic dimensions, and the inequality of intergroup allocation of physicians tended to increase in the demographic dimension. This phenomenon may be caused by the following reasons. First, the incentive system for primary care physicians, such as salary and promotion, is inadequate, which results in a lower willingness of physicians to work in township medical institutions. Physicians have a greater willingness to work in developed cities and large medical institutions, which leads to a greater difference in the allocation of human resources between urban and rural areas [36, 37]. Second, Yunnan and Tibet are located at higher altitudes and are oxygen-deprived, making it more difficult for physicians and nurses to adapt. Third, township medical institutions are usually located in rural areas, and most rural areas are ethnic minority areas that have a strong traditional culture and are more backward in economic conditions and working environment. These factors make it harder for physicians and nurses to integrate into these areas and gain a sense of identity and belonging, which limits the introduction of professional talent and makes human resources unequally distributed within provinces. Therefore, the government should optimize the practice environment, strengthen incentive mechanisms, widen promotion channels, encourage more physicians and nurses to work in township medical institutions, pay attention to humanistic care for physicians and nurses, and help them integrate into local life to ultimately improve the equity of human resource allocation.

Most previous studies of resource allocation in China have focused on the whole country or a specific province or city, but few studies have paid attention to one region. In addition, health resources in Western China are poorer than those in Eastern and Central China, and the inequity problem of Western China may be more prominent, but few studies have investigated the issue. Therefore, this study supplements the analysis of health resource allocation in Western China and provides a reference for policy makers in the Chinese government. In addition, after the COVID-19 outbreak in late 2019, the different COVID-19 prevalence and the different medical needs in each province may lead the changes of health resource allocation and researchers may start to think about how to optimize health resource allocation in Western China during the epidemic period and post-epidemic period. This study can provide preliminary data and a research basis for their future studies.

However, the present study has several limitations. First, based on the *13th Five-Year Plan*, the indexes of the medical and health service system also include the number of general physicians and the proportion of beds in socially run hospitals in the total number of hospital beds. In addition, the efficiency of health resource use is a key factor affecting health equity and the capacity of medical institutions to provide services [37]. However, due to the limited availability and integrity of the data collected, the above problems were not further analysed in this paper. Second, medical needs refer to whether people can easily access the health services they need and are able to pay for them. The satisfaction of this need is one of the indicators of the accessibility of health resources [38, 39]. Although this study found a gradual improvement in the equity of health resource allocation in Western China from 2014 to 2018, it is not yet possible to measure improvements in medical needs based on the methodology used in this study and the data currently available.

## Conclusion

Based on the research above, we found that from 2014 to 2018, the total amount of health resources in Western China increased as a whole, and the government’s measures to improve resource equity played a role. However, the inequity of health resource distribution persisted in Western China. The major differences in health resource allocation existed in the geographic dimension and the intragroup dimension, which may impact health resource accessibility. Moreover, the equity of human resources allocation was relatively poor, which warrants urgent promotion. These problems are also key factors related to people’s health. Hence, the government should pay more attention to the allocation of health resources in Western China and solve these problems in various ways, such as by increasing policy implementation, enhancing financial investment, establishing internet hospitals, and strengthening humanistic care to improve the equity of health resource allocation.

## Data Availability

The data in this paper obtained from China statistical yearbook from 2015 to 2019, and the public data which released by the National Health and Family Planning Commission from 2015 to 2019.

## Abbreviations

13th Five-Year Plan: China's 13th Five-Year Plan for Health and Wellness
WHO: World Health Organization
